# Proteomic Analysis Reveals the Positive Roles of the Plant-Growth-Promoting Rhizobacterium NSY50 in the Response of Cucumber Roots to Fusarium *oxysporum* f. sp. *cucumerinum* Inoculation

**DOI:** 10.3389/fpls.2016.01859

**Published:** 2016-12-14

**Authors:** Nanshan Du, Lu Shi, Yinghui Yuan, Bin Li, Sheng Shu, Jin Sun, Shirong Guo

**Affiliations:** ^1^Key Laboratory of Southern Vegetable Crop Genetic Improvement in Ministry of Agriculture, College of Horticulture, Nanjing Agricultural UniversityNanjing, China; ^2^Department of Horticulture, Shanxi Agricultural UniversityTaigu, China; ^3^Suqian Academy of Protected Horticulture, Nanjing Agricultural UniversitySuqian, China

**Keywords:** cucumber, *Fusarium oxysporum* f. sp. *cucumerinum*, plant-growth-promoting rhizobacteria, proteomics, root

## Abstract

Plant-growth-promoting rhizobacteria (PGPR) can both improve plant growth and enhance plant resistance against a variety of environmental stresses. To investigate the mechanisms that PGPR use to protect plants under pathogenic attack, transmission electron microscopy analysis and a proteomic approach were designed to test the effects of the new potential PGPR strain *Paenibacillus polymyxa* NSY50 on cucumber seedling roots after they were inoculated with the destructive phytopathogen *Fusarium oxysporum* f. sp. *cucumerinum* (FOC). NSY50 could apparently mitigate the injury caused by the FOC infection and maintain the stability of cell structures. The two-dimensional electrophoresis (2-DE) approach in conjunction with MALDI-TOF/TOF analysis revealed a total of 56 proteins that were differentially expressed in response to NSY50 and/or FOC. The application of NSY50 up-regulated most of the identified proteins that were involved in carbohydrate metabolism and amino acid metabolism under normal conditions, which implied that both energy generation and the production of amino acids were enhanced, thereby ensuring an adequate supply of amino acids for the synthesis of new proteins in cucumber seedlings to promote plant growth. Inoculation with FOC inhibited most of the proteins related to carbohydrate and energy metabolism and to protein metabolism. The combined inoculation treatment (NSY50+FOC) accumulated abundant proteins involved in defense mechanisms against oxidation and detoxification as well as carbohydrate metabolism, which might play important roles in preventing pathogens from attacking. Meanwhile, western blotting was used to analyze the accumulation of enolase (ENO) and S-adenosylmethionine synthase (SAMs). NSY50 further increased the expression of ENO and SAMs under FOC stress. In addition, NSY50 adjusted the transcription levels of genes related to those proteins. Taken together, these results suggest that *P. polymyxa* NSY50 may promote plant growth and alleviate FOC-induced damage by improving the metabolism and activation of defense-related proteins in cucumber roots.

## Introduction

Cucumber (*Cucumis sativus* L.) is an important and popular vegetable cash crop that is consumed worldwide. However, the production of this plant is severely threatened by cucumber *Fusarium* wilt, which is caused by the soilborne fungal pathogen *Fusarium oxysporum* f. sp. *cucumerinum* (FOC), a destructive vascular disease that can cause plant death and serious economic loss (Ahn et al., 1998; Huang et al., 2012). Because there are no available effective chemical products or resistant varieties and because grafting is time consuming (Chung et al., 2008; Cao et al., 2012), other effective, economical, and environmentally friendly cultivation methods have been developed and tested.

To date, alternative methods, such as biological control agents, have been shown to be effective and have been increasingly applied in the field (Yang et al., 2010; Li et al., 2012; Lin et al., 2014; Xu et al., 2014). Among these methods, some beneficial bacteria inhabiting the plant rhizosphere, called plant growth-promoting rhizobacteria (PGPR), are directly or indirectly involved in promoting plant growth and the biological control of plant diseases (Kloepper and Metting, 1992). A variety of mechanisms have been confirmed to improve plant health and increase crop productivity for those strains, such as synthesizing different antimicrobial compounds (Tamehiro et al., 2002; Ongena et al., 2007; Chen et al., 2014), various hormones (Bottini et al., 2004; Martínez-Viveros et al., 2010; Kochar et al., 2011), increasing the availability of plant nutrients in the rhizosphere (Idriss et al., 2002; Palacios et al., 2014), and inducing systemic resistance in host plants (Niu et al., 2011; Jiang et al., 2015).

Numerous technical methods, such as DNA microarray technology and proteomic analysis, have been applied to improve our understanding of plant-PGPR interactions. A study in the *Arabidopsis* root transcriptome showed that extensive changes were induced in hormone- and defense-related genes after exposure to *Azospirillum brasilense* Sp245 (Spaepen et al., 2014). In another study, the ISR- and iron acquisition-related transcription factors in *Arabidopsis* roots were activated by *Pseudomonas fluorescens* WCS417 and its volatiles (Zamioudis et al., 2015). A dynamic protein network was designed to explain the mechanism of *P. putida* UW4 to release hypoxic stress and promote cucumber growth (Li et al., 2013b). Proteins involved in growth promotion and defense reactions were significantly induced in *Arabidopsis* following inoculation with *Paenibacillus polymyxa* E681 (Kwon et al., 2016). In addition, many other researchers have used proteomics to investigate plant-pathogen interactions. Wang et al. (2011) found that a switch from glycolysis to the pentose phosphate pathway may be an active response of the cotton plant against *V. dahliae* infection to enhance wilt tolerance or resistance. Total root protein was isolated from infected cucumber roots of the susceptible bulk and resistant bulk of cucumber generation F2, and the result showed that jasmonic acid and redox signaling components occurred in response to *F. oxysporum* infection in resistant plants (Zhang et al., 2016). Eighty-six differentially expressed proteins were identified from the leaves of resistant tomato cultivar “Zheza-301” and susceptible cultivar “Jinpeng-1” after TYLCV infection, which demonstrated that an interaction network between tomato leaves and TYLCV infection was established (Huang et al., 2016). To date, although several papers have addressed the use of proteomics to investigate the interactions of plants and pathogens (Konishi et al., 2001; Wang et al., 2011; Carrillo et al., 2014) or plants and PGPR (Kandasamy et al., 2009; Wang et al., 2013), little research is available on the effects of PGPB on cucumber plants under pathogenic attack.

In this present study, a promising bio-control agent *Paenibacillus polymyxa*-NSY50, which was originally isolated from the high suppression capacity of compost (Du et al., 2015; Shi et al., 2016), was used to better understand direct or indirect interactions among *P. polymyxa* NSY50, the pathogen FOC and cucumber roots. A total of 56 proteins were identified that were up- or down-regulated by the inoculation of *P. polymyxa* NSY50 and/or FOC, and the proteins could be classified into multiple biological functions. This work provides key insights into the promotion mechanisms of *P. polymyxa* NSY50 action to determine the optimal use of this beneficial PGPR in sustainable agricultural practices.

## Material and methods

### Plant material, microbial culture conditions, and treatments

Cucumber (*Cucumis sativus* L. cv. Jinchun No. 2) seeds were sown in quartz sand and cultivated in a greenhouse. *Paenibacillus polymyxa*-NSY50 was grown on LB medium at 28°C for 3 days, and the cucumber Fusarium wilt pathogen *F. oxysporum* f. sp. *cucumerinum* (FOC) was incubated in PDA liquid culture for 7 days.

At the two true leaves stage, the cucumber seedlings were transplanted to tanks containing half-strength Hoagland nutrient solution (He et al., 2015). After 3 days of pre-culture, the seedlings were treated as follows: (1) control, control plants were grown in Hoagland's solution; (2) NSY50, seedlings grown in Hoagland nutrient solution containing 500-mL 1.0 × 10^8^ NSY50 cell suspension; (3) FOC: 500 mL of a cell suspension of FOC at 1 × 10^7^ conidia/mL was poured into the tanks after 6 days of culture; and (4) NSY50+FOC: plants were inoculated with 500 mL of NSY50 (1 × 10^8^ CFU/mL) after 3 days of culture, and 3 days later, the plants were challenge-inoculated with 500 mL of a cell suspension of FOC (1 × 10^7^ conidia/mL). The experiment was arranged in a randomized complete block design with three replicates for each treatment, which resulted in a total of 36 seedlings per treatment. Seedlings were reared in a greenhouse at 25–30°C in the daytime and at 15–18°C at night under natural lighting at 60–75% relative humidity.

### Plant growth analysis

After 9 days of FOC treatment, shoot, and root were separated from one another and measured after being washed with sterile distilled water. The plant height, biomass, and volume of root in each treatment were measured as previously described (Shi et al., 2016). Each data point was the average of measurements collected in triplicate.

### TEM analysis

For transmission electron microscopy, roots of seedlings from different treatments were excised and immediately cut into small segments (1~2 mm, including the tip); The samples were then fixed with 2.5% glutaraldehyde in 0.1 M phosphate buffer (pH 7.4) for 24 h (primary fixation) and immersed in 2% osmic acid in the same buffer for 2 h (second fixation). After dehydration in acetone and embedding in Durcupan ACM (Fluka), the resulting roots were cut to obtain ultra-thin sections, stained with uranium acetate and lead citrate in series and examined using a HITACHI transmission electron microscope (Carl Zeiss, Göttingen, Germany) at an accelerating voltage of 80 kV.

### Protein extraction

The cucumber roots were prepared for protein fractionation 3 days post-inoculation of FOC. Total protein extraction was performed using a trichloroacetic acid, i.e., the acetone precipitation method modified described by Hurkman and Tanaka (1986). Fresh root samples (2 g) were milled with liquid nitrogen and suspended in extraction buffer as described by An et al. (2016). The homogenate was centrifuged at 15,000 g at 4°C for 20 min. An aliquot (1 mL) of the resulting supernatant was transferred to a new tube and precipitated with ice-cold acetone (10% TCA and 0.07% β-mercaptoethanol) overnight at −20°C, and then the resulting protein sample was centrifuged at 20,000 g for 25 min. The pellet was washed three times with cold acetone (0.07% β-mercaptoethanol) and allowed to stand at −20°C for 2 h. Finally, the protein pellet was air-dried and used for 2-DE.

### 2-DE and image analysis

Isoelectric focusing (IEF) was performed using an 18-cm IPG linear gradient strip, pH 4–7 (GE Healthcare, San Francisco, USA). The dried protein pellet was rehydrated in a rehydration buffer that contained 7 M urea, 2 M thiourea, 4% 3-[(3-cholanidopropyl) dimethylammonio]-1-propanesulfonic acid (w/v), 40 mM DTT, 0.5% (v/v) IPG buffer 4–7 and 0.01% (w/v) bromophenol blue. The protein levels were quantified using the Bradford method (Bradford, 1976). The 350 μL protein samples were loaded on IPG strips containing 800 μg of protein and rehydrated for 12–16 h at 25°C. After rehydration, the IPG strips were run on an Ettan IPGphor 3 (GE Healthcare, San Francisco, USA) using the following protocol: the voltage for IEF was set at 100 V for 1 h, followed by 200 V for 1 h, 200 V for 1 h, 500 V for 1 h, 1000 V for 1 h, 4000 V for 1 h, a gradient of 10,000 V for 1 h, and then a 10,000 V rapid focus, which reached a total of 75,000 V h with a maximum electric current of 50 μA per strip. After accomplishing the first dimension, IEF strips were equilibrated for 15 min in a 2D equilibrium buffer that consisted of 6 M urea, 30% glycerol (v/v), 50 mM Tris-HCl (pH 8.8), and 2% sodium dodecyl sulfate (SDS) containing 1% DTT (w/v); the strips were then incubated in 2.5% (w/v) iodoacetamide instead of DTT for 15 min. Then, the strips were placed directly onto 12.5% polyacrylamide-SDS slab gels and sealed using 1% molten agarose solution. The second dimensional separation was conducted using the EttanDalySix electrophoresis system (GE Healthcare, USA). At 15 mA per gel, the electrophoresis was not stopped until the bromophenol blue dye reached approximately 1 cm from the bottom of the gel. Protein spots were visualized using Coomassie Brilliant Blue (CBB) R-250.

The CBB-stained 2-DE gels were scanned using an Image scanner III (GE Healthcare, USA). The digitized images of three independent experiments were analyzed with Imagemaster 2D Platinum version 5.0 (GE Healthcare, USA). The abundance of each protein spot was estimated by the percentage volume (vol.%), and the spot volumes were normalized as the ratio of the total volume for all of the spots that were present. Spots with a vol.% that represented at least a 1.5-fold change and was significant at the *P* < 0.05 level (Duncan's multiple range tests) were used for identification.

### Protein identification and functional classification

The differentially expressed protein spots were extracted from CBB-stained preparative polyacrylamide gels and then identified using an ABI 5800 Proteomics Analyzer MALDI-TOF/TOF (Applied Biosystems, Foster City, CA, USA). The data lists were used as a query to search in the NCBI (http://www.ncbi.nlm.nih.gov/) and cucumber genomics databases (http://cucumber.genomics.org.cn) using the software MASCOT version 2.2 (Matrix Science, London, UK). The following search parameter criteria were used: trypsin cleavage, one missed cleavage site allowed; carbamidomethyl set as a fixed modification; oxidation of methionines allowed as a variable modification; a peptide mass tolerance within 100 ppm; fragment tolerance set to ± 0.4 Da; and a minimum ion score confidence interval for MS/MS data set to 95%.

Identified proteins were categorized according to the biological processes with which they were involved based on Gene Ontology (GO) (http://www.geneontology.org/) and the Uniprot Protein Knowledgebase (http://www.uniprot.org/). Hierarchical clustering of the protein expression patterns was performed on the log-transformed (using log base 2) spot abundance ratios using the software Cluster version 3.0. A heat map was created using Java Treeview.

### Western blot analysis

Root tissues were milled in a mortar with ice-cold extraction buffer containing 30 mM Tris—HCl (pH 8.7), 1 mM MgCl_2_, 0.7 M saccharose, 1 mM EDTA, 1 mM DTT, 1 mM PMSF, and 1 mM ascorbic acid. The extracted protein was quantified using the Bradford method (1976), denatured at 95°C for 3–5 min and then stored at −20°C until analysis.

For western blot analysis, SDS-polyacrylamide gel electrophoresis (SDS-PAGE) was conducted using the methods of Laemmli (1970). Briefly, 11 μg protein samples were transferred to a 0.45 μm PVDF membrane at 10 V for 1.5 h and were washed with TBST three times; then, the PVDF membrane was blocked with 5% nonfat dry milk for 2 h, washed with TBST three times, and incubated with monoclonal antibodies against S-adenosylmethionine synthase and enolase (produced in rabbit; Univ-bio, Shanghai, China) for 2 h. The membrane was washed with TBST and incubated at room temperature for 1 h with a Goat Anti-Rabbit IgG HRP-conjugate. The membrane was then washed with TBST three times and developed using diaminobenzidene (DAB) and H_2_O_2_ (He et al., 2012).

### Total RNA extraction and quantitative real-time PCR (qRT-PCR) analysis

Root tissues were harvested at 1, 3, 9 days after inoculation with FOC. The total RNA was extracted according to the TRI reagent protocol (Takara Bio Inc.) and then converted into cDNA following the manufacturer's instructions. Primers were designed based on the sequences obtained from NCBI using Beacon Designer 7.90 (Supplementary Table 1). qRT-PCR was performed using the SYBR PrimeScript™ RT-PCR Kit (Takara Bio Inc.) in accordance with the manufacturer's instructions and run on a StepOne™ real-time PCR system (Applied Biosystems, Singapore). The relative gene expression was calculated using the 2^−ΔΔCt^ method (Livak and Schmittgen, 2001), and the methods were based on a previously described protocol (Shi et al., 2016). All reactions were conducted with three biological replicates.

### Statistical analysis

All data were statistically analyzed using SPSS 20.0 for Windows, and significance was assigned at the *P* < 0.05 level using Duncan's multiple comparisons test.

## Results

### Plant growth

The biomass of the cucumber seedlings was measured 9 days post-inoculation (dpi) with the pathogen. The plant height, shoot freshness and dry mass of NSY50-treated seedlings were significantly greater than those of plants grown under control conditions (Table 1). However, pathogen inoculation (treatment FOC) seriously decreased the growth of the cucumber seedlings, the plant height, shoot fresh weight, dry weight and root volume and the root fresh weight and dry weight, which were significantly suppressed compared to the control. Moreover, under pathogen stress, plant biomass was significantly higher in *P. polymyxa* NSY50 pre-treated cucumber plants, which showed a 1.2–1.5-fold up-regulation in plant growth compared to untreated plants. Thus, the results clearly indicated that the pathogen inoculation inhibited growth in cucumber seedlings and that *P. polymyxa* NSY50 can release at least a portion of the stress and promote plant growth.

**Table 1:** **Effects of plant-growth-promoting bacteria on the growth of cucumber seedlings under Fusarium wilt stress**.

**Treatment**	**Plant height (cm)**	**Shoot fresh mass (g/plant)**	**Shoot dry mass (g/plant)**	**Root fresh mass (g/plant)**	**Root dry mass (g/plant)**	**Root volume (cm^3^)**
Control	20.97 ± 1.87b	10.08 ± 0.40b	0.77 ± 0.07b	2.82 ± 0.10a	0.15 ± 0.009a	2.14 ± 0.20a
NSY50	26.07 ± 1.01a	14.23 ± 0.47a	0.96 ± 0.02a	2.78 ± 0.15a	0.13 ± 0.006ab	2.05 ± 0.09a
FOC	16.53 ± 1.26c	7.16 ± 0.96c	0.45 ± 0.05c	1.53 ± 0.12c	0.08 ± 0.013c	1.17 ± 0.09c
NSY50+FOC	19.07 ± 1.07b	9.37 ± 0.94b	0.63 ± 0.05b	2.28 ± 0.18b	0.11 ± 0.009b	1.59 ± 0.10bc

*Each value is the mean ± SE of three replicates. Different letters indicate significant differences at P < 0.05 according to Duncan's multiple range tests. Control, seedlings cultured in normal nutrient solution; NSY50, control+ P. polymyxa NSY50 (2.5 × 106 CFU/mL); FOC, control+ FOC (2.5 × 105 conidia/mL); NSY50+FOC, pre-treated with NSY50 (2.5 × 106 CFU/mL) for 3 days, then challenged with FOC (2.5 × 105 conidia/mL)*.

### Ultrastructure of the roots

**Figure 1:**
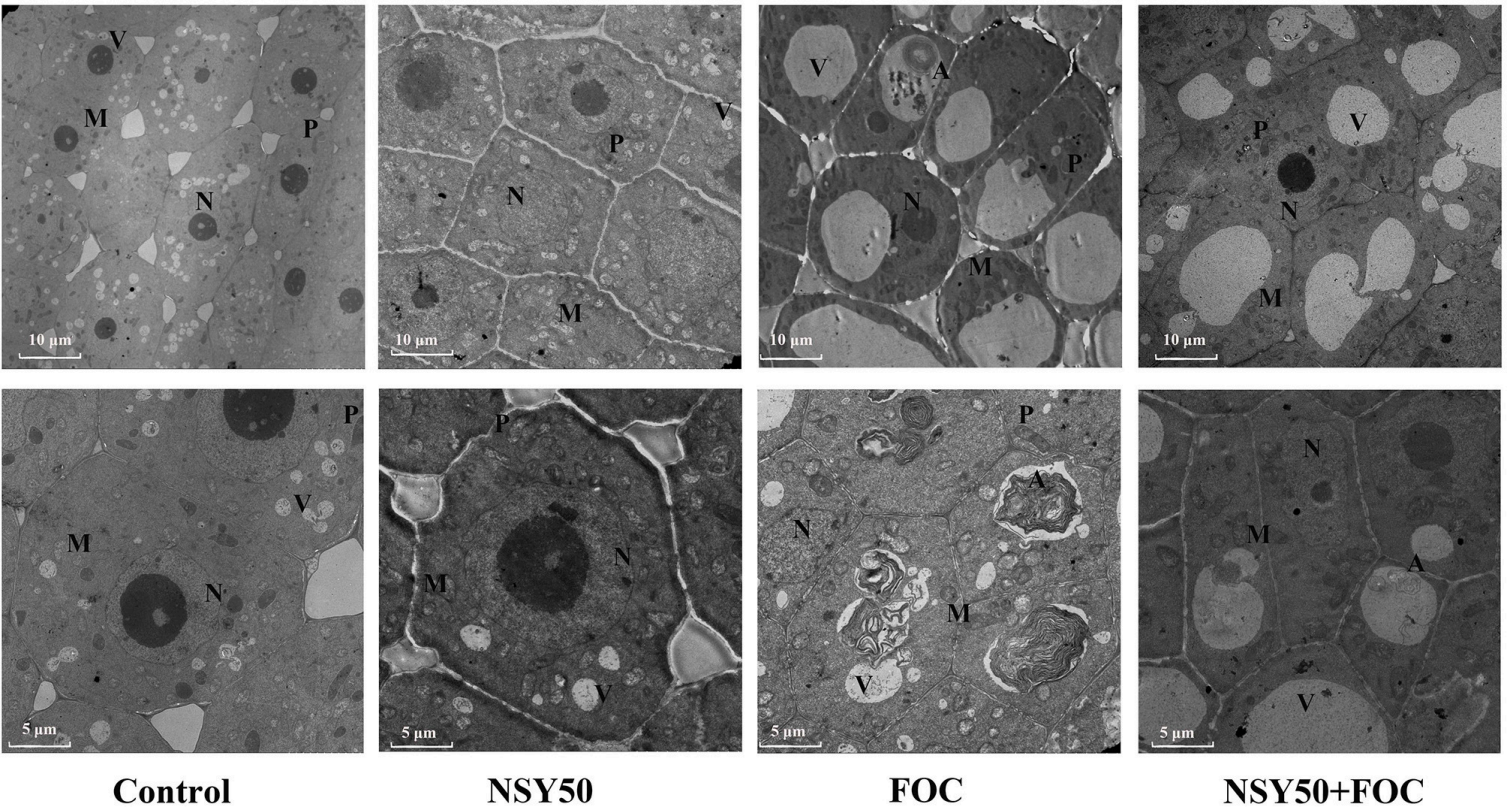
**Effects of NSY50 on the cellular ultrastructure of cucumber root tips with or without FOC inoculation 3 days after treatment**. Samples comprised 3 mm root tips. Control, seedlings cultured in normal nutrient solution; NSY50, control+ *P. polymyxa* NSY50 (2.5 × 10^6^ CFU/mL); FOC, control+ FOC (2.5 × 10^5^ conidia/mL); NSY50+FOC, pre-treated with NSY50 (2.5 × 10^6^ CFU/mL) for 3 days, then challenged with FOC (2.5 × 10^5^ conidia/mL).M, Mitochondria; N, Nucleus; V, Vacuole; P, Plastid; A, Autophagosome.

Under control conditions, the vacuoles had small sizes, and many nuclei and mitochondria could be seen clearly under the microscope (Figure 1). The cellular ultrastructure of cucumber roots seemed to show no difference between the control plants and plants treated with NSY50 alone (NSY50). However, the inoculation of FOC (FOC) caused remarkable structural changes. Specifically, the karyotheca appeared to have contracted, and the number of the mitochondria decreased compared to the control. Furthermore, the number of central vacuoles and autophagosomes appeared to indicate that the pathogen attack advanced the cell aging and death process. Pre-treatment with NSY50 (NSY50+FOC) apparently enhanced the stability of the cell structure.

### Identification and functional classification of proteins

**Figure 2:**
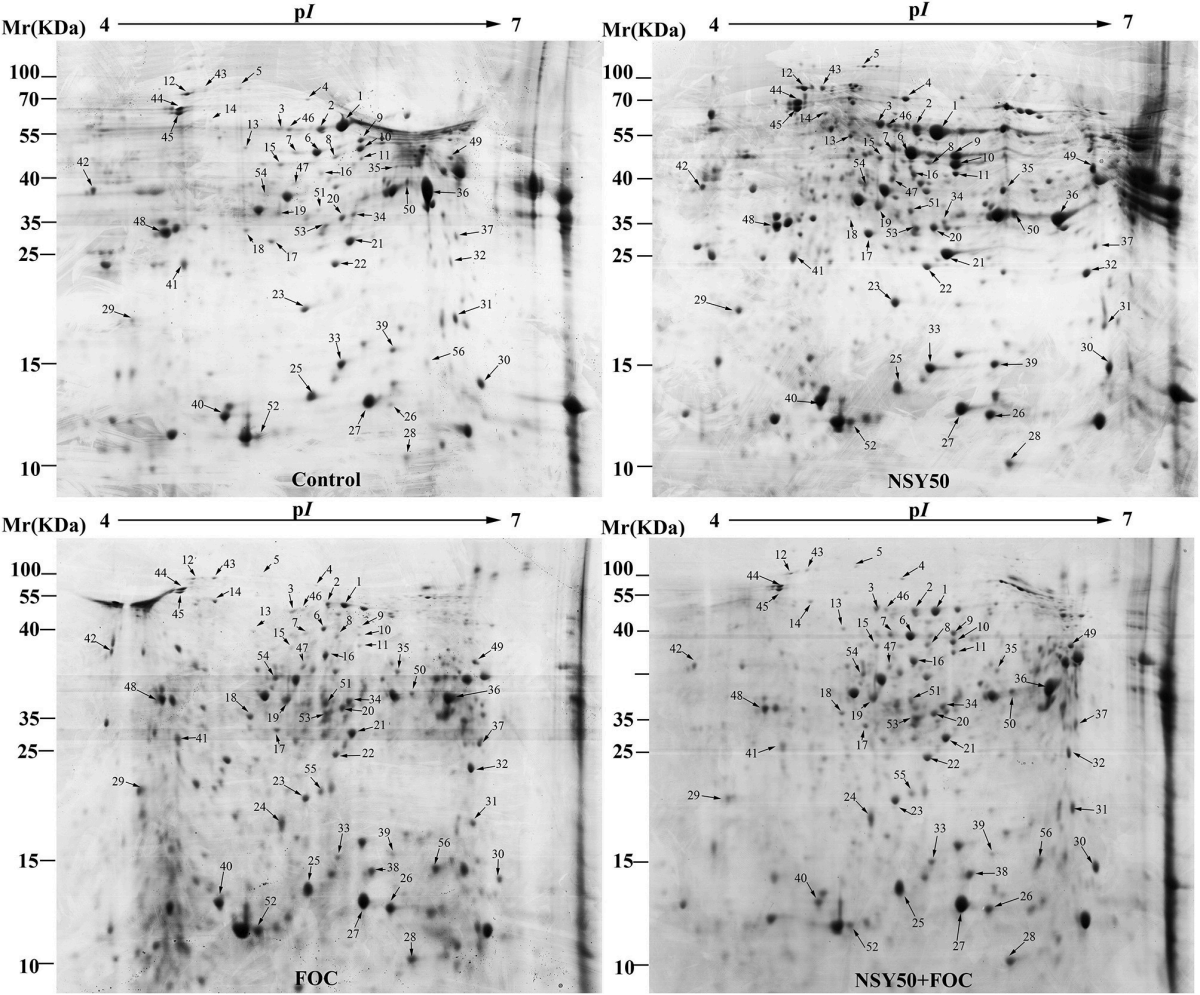
**Representative 2-DE gel images of total protein extracts from root samples inoculated with NSY50 and/or FOC**. An equal amount (800 μg) of total proteins were separated by IEF/SDS-PAGE and then stained with Coomassie Brilliant Blue (R-250) and loaded on each 18 cm gel strip (pH 4–7, linear). The numbers for the 56 differentially expressed proteins are marked and annotated according to the numbering in Table 2. Control, seedlings cultured in normal nutrient solution; NSY50, control+ *P. polymyxa* NSY50 (2.5 × 10^6^ CFU/mL); FOC, control+ FOC (2.5 × 10^5^ conidia/mL); NSY50+FOC, pre-treated with NSY50 (2.5 × 10^6^ CFU/mL) for 3 days then challenged with FOC (2.5 × 10^5^ conidia/mL).

Approximately 350 reproducible protein spots were detected on the 2-DE gels, and 56 differentially expressed protein spots (changes ≥ 1.5-fold) were successfully identified by MALDI-TOF/TOF MS (Figure 2; Supplementary Figure 1). These differentially expressed proteins are listed in Table 2.

**Table 2:** **Root proteins responsive to NSY50 and/or FOC identified by MALDI-TOF/TOF MS**.

**Spot no.^a^**	**Protein name**	**NCBI Accession no**.	**Mr(kDa)/PI**		**MP^b^**	**Score**	**Cov^c^ (%)**	**Ratio^d^**			
			**Theoretical**	**Experimental**				**Control/Control**	**NSY50/Control**	**FOC/Control**	**NSY50+FOC/FOC**
**CARBOHYDRATE AND ENERGY METABOLISM (11)**											
**Glycolysis**											
1	Enolase isoform X2	gi|778717375	43.13/5.80	60.0/5.78	6	202	26.13	1.00	0.94	0.18	1.55
2	Enolase isoform X2	gi|778717375	43.13/5.80	61.25/5.67	15	326	59.30	1.00	0.70	0.37	1.51
13	Protein DJ-1 homolog D	gi|449469102	42.40/5.12	53.0/5.30	10	187	37.95	1.00	2.28	0.21	4.25
17	Enolase isoform X1	gi|449451102	47.84/5.48	33.0/5.42	17	427	34.91	1.00	0.94	0.18	4.05
18	Enolase isoform X1	gi|449451102	47.94/5.48	33.0/5.42	2	137	5.18	1.00	0.57	1.27	0.45
20	Probable fructokinase-4	gi|449454574	35.79/5.62	36.75/5.48	21	531	61.63	1.00	1.18	0.60	1.36
46	Enolase isoform X2	gi|778717375	43.13/5.80	60.75/5.51	14	292	49.50	1.00	2.58	0.86	1.66
55	Fructose-bisphosphate aldolase, cytoplasmic isozyme-like	gi|449451108	38.73/7.57	23.50/6.65	18	293	51.96	0.00	0.00	0.15	0.62
**Tricarboxylic Acid Cycle**											
36	Malate dehydrogenase, mitochondrial	gi|449438883	36.41/8.52	37.25/6.33	7	201	32.28	1.00	0.52	0.24	2.34
**Sucrose Metabolism**											
43	Acid beta-fructofuranosidase-like	gi|449451749	69.80/4.92	90.25/5.11	9	17	15.56	1.00	3.25	2.57	0.61
**Energy Metabolism**											
3	ATP synthase subunit beta, mitochondrial-like	gi|449465916	60.18/5.90	61.40/5.48	30	662	70.89	1.00	5.56	0.60	3.44
**PROTEIN METABOLISM (14)**											
**Protein Folding and Assembly**											
14	Protein disulfide-isomerase	gi|449464162	57.27/4.88	65.0/5.13	19	209	53.92	1.00	1.90	2.42	0.45
23	20 kDa chaperonin, chloroplastic	gi|778663199	26.87/7.85	27.8/5.73	9	183	43.36	1.00	1.04	0.56	1.26
42	Protein disulfide-isomerase	gi|449464162	57.27/4.88	41.75/4.53	18	122	52.94	1.00	0.80	0.37	0.72
44	Protein disulfide-isomerase	gi|449464162	57.27/4.88	72.0/4.97	29	622	64.12	1.00	0.75	0.42	0.87
45	Protein disulfide-isomerase	gi|449464162	57.27/4.88	69.25/4.97	33	911	72.35	1.00	1.13	0.60	0.94
**Protein Biosynthesis**											
33	Eukaryotic translation initiation factor 5A	gi|449455523	17.64/5.60	18.00/5.67	8	82	61.64	1.00	0.85	0.13	2.58
38	Eukaryotic translation initiation factor 5A	gi|449455523	17.64/5.60	17.0/5.92	10	185	54.72	0.00	0.00	0.12	1.88
39	Eukaryotic translation initiation factor 5A	gi|449455523	17.64/5.60	18.67/6.07	13	191	73.58	1.00	1.18	0.15	1.28
47	Elongation factor 2	gi|778713730	95.03/5.97	41.25/5.55	13	113	19.69	1.00	2.70	1.35	0.65
56	Eukaryotic translation initiation factor 5A-2	gi|700210064	17.77/5.59	18.0/6.28	4	104	22.50	1.00	0.00	4.26	1.36
**Protein Degradation**											
22	Proteasome subunit alpha type-2-A	gi|449455401	25.63/5.51	31.0/5.83	3	74	13.44	1.00	1.48	0.44	1.54
41	Thiol protease aleurain-like	gi|449452572	39.55/6.26	29.0/4.97	11	423	49.44	1.00	1.58	0.59	0.89
**Protein Transport**											
28	Nuclear transport factor 2-like	gi|778689955	13.67/6.00	12.25/6.15	7	351	95.93	1.00	5.13	4.58	1.14
**Protein Modification**											
30	Ubiquitin-conjugating enzyme E2 variant 1C	gi|449437446	16.68/6.20	17.0/6.59	16	319	85.62	1.00	1.19	0.37	2.03
**DEFENSE RESPONSE (16)**											
**Antioxidant Reaction**											
19	Peroxidase 2-like	gi|778693042	35.94/5.51	35.0/5.29	8	106	26.06	1.00	4.81	3.95	1.61
21	L-ascorbate peroxidase, cytosolic-like	gi|525507192	27.55/5.43	35.25/5.77	14	318	67.87	1.00	1.55	0.57	1.01
24	Peroxidase 2-like	gi|778693034	37.17/5.51	21.0/5.45	8	365	23.37	0.00	0.00	0.13	1.58
25	Superoxide dismutase [Cu-Zn], chloroplastic	gi|449456060	22.72/5.87	16.0/5.59	8	557	73.09	1.00	0.59	0.63	1.51
27	Superoxide dismutase [Cu-Zn]-like isoform X1	gi|778655163	15.48/5.43	15.4/5.90	1	137	13.82	1.00	0.69	0.89	2.01
32	Glutathione S-transferase-like	gi|778727975	24.00/5.98	27.60/6.46	12	156	68.37	1.00	2.33	1.34	0.82
37	Glutathione S-transferase DHAR2	gi|778700922	23.90/6.18	32.4/6.50	5	105	32.86	1.00	1.04	0.56	1.66
50	Probable aldo-keto reductase 4	gi|778689965	37.89/5.78	37.75/6.15	21	223	61.70	1.00	2.14	0.68	2.81
52	Thioredoxin H-type 1 isoform X2	gi|778671470	13.74/5.91	14.0/5.33	6	119	36.59	1.00	1.67	2.72	0.82
53	Probable L-ascorbate peroxidase 6, chloroplastic isoform X2	gi|778715658	45.03/7.09	34.75/5.68	13	189	40.44	1.00	1.67	2.17	0.43
54	Peroxidase 2-like	gi|778693034	37.17/5.51	39.74/5.40	12	273	47.04	0.00	0.03	0.11	0.78
**Other Defense Response**											
4	Heat shock 70 kDa protein, mitochondrial	gi|449459554	73.25/5.69	78.5/5.61	25	269	44.12	1.00	3.31	0.37	1.45
12	Heat shock protein 70	gi|1143427	75.48/5.15	87.75/5.02	24	300	39.04	1.00	1.26	0.75	0.52
26	MLP-like protein 328	gi|449449064	17.66/5.65	15.0/6.04	12	347	87.42	1.00	3.23	3.68	0.85
31	Glycine-rich protein 2	gi|778722923	20.01/6.29	21.75/6.48	5	64	45.28	1.00	0.50	0.70	0.95
40	Major allergen Pru ar 1-like	gi|778714676	17.27/4.98	15.25/5.16	15	704	84.28	1.00	1.77	0.93	0.41
**AMINO ACID METABOLISM (10)**											
6	S-adenosylmethionine synthase 2	gi|778728392	43.65/5.35	51.25/5.65	23	260	86.01	1.00	1.98	0.22	4.15
7	S-adenosylmethionine synthase 2-like	gi|449472803	44.67/5.29	51.50/5.55	18	301	71.07	1.00	0.94	0.29	1.92
8	S-adenosylmethionine synthase 2	gi|778728392	43.65/5.35	48.75/5.74	18	470	63.36	1.00	0.76	0.37	1.29
9	S-adenosylmethionine synthase 4	gi|449451048	43.03/6.07	53.25/5.87	13	301	36.15	1.00	2.21	0.08	9.54
10	S-adenosylmethionine synthase 4	gi|449451048	43.03/6.07	49.75/5.87	15	329	48.72	1.00	1.55	0.15	3.61
11	Glutamine synthetase leaf isozyme, chloroplastic	gi|778678626	48.03/7.62	46.25/5.87	18	146	51.85	1.00	2.05	0.80	1.88
15	S-adenosylmethionine synthase 2-like	gi|449472803	44.67/5.29	47.25/5.48	18	277	56.61	1.00	0.99	0.29	2.51
16	S-adenosylmethionine synthase 2	gi|778728392	43.65/5.35	43.25/5.67	16	314	58.27	1.00	1.01	1.72	1.26
35	Glutamine synthetase cytosolic isozyme-like	gi|525507210	39.42/5.82	42.5/6.07	9	71	32.02	1.00	5.96	2.27	1.24
48	S-adenosylmethionine synthase 2-like	gi|449472803	44.67/5.29	35.20/4.88	11	137	45.14	1.00	0.65	0.47	0.74
**FATTY ACID METABOLIS (2)**											
49	12-oxophytodienoate reductase 1	gi|778672814	42.31/6.17	45.80/6.47	21	252	56.12	1.00	2.68	1.33	0.96
51	Enoyl-[acyl-carrier-protein] reductase [NADH], chloroplastic-like	gi|449448774	41.67/8.64	36.50/5.66	13	446	52.94	1.00	1.10	3.08	0.99
**SECONDARY METABOLISM (1)**											
34	Nitrile-specifier protein 5	gi|449444472	35.62/5.30	36.25/5.82	19	206	48.46	1.00	0.54	1.43	0.64
**CELL RELATED PROTEIN (2)**											
5	Cell division control protein 48 homolog E	gi|449440119	90.09/5.06	102/5.30	32	274	49.75	1.00	0.72	0.20	0.92
29	Translationally-controlled tumor protein homolog	gi|449432858	18.76/4.56	22.50/4.71	9	176	67.26	1.00	1.73	4.19	0.20

a *Spot number corresponding with 2-DE gel as shown in Figure 2*.

b *Number of identified peptides*.

c *Percentage of sequence coverage by matched peptides*.

d *The values higher than 1.5 or lower than 0.67 indicate significant changes*.

**Figure 3:**
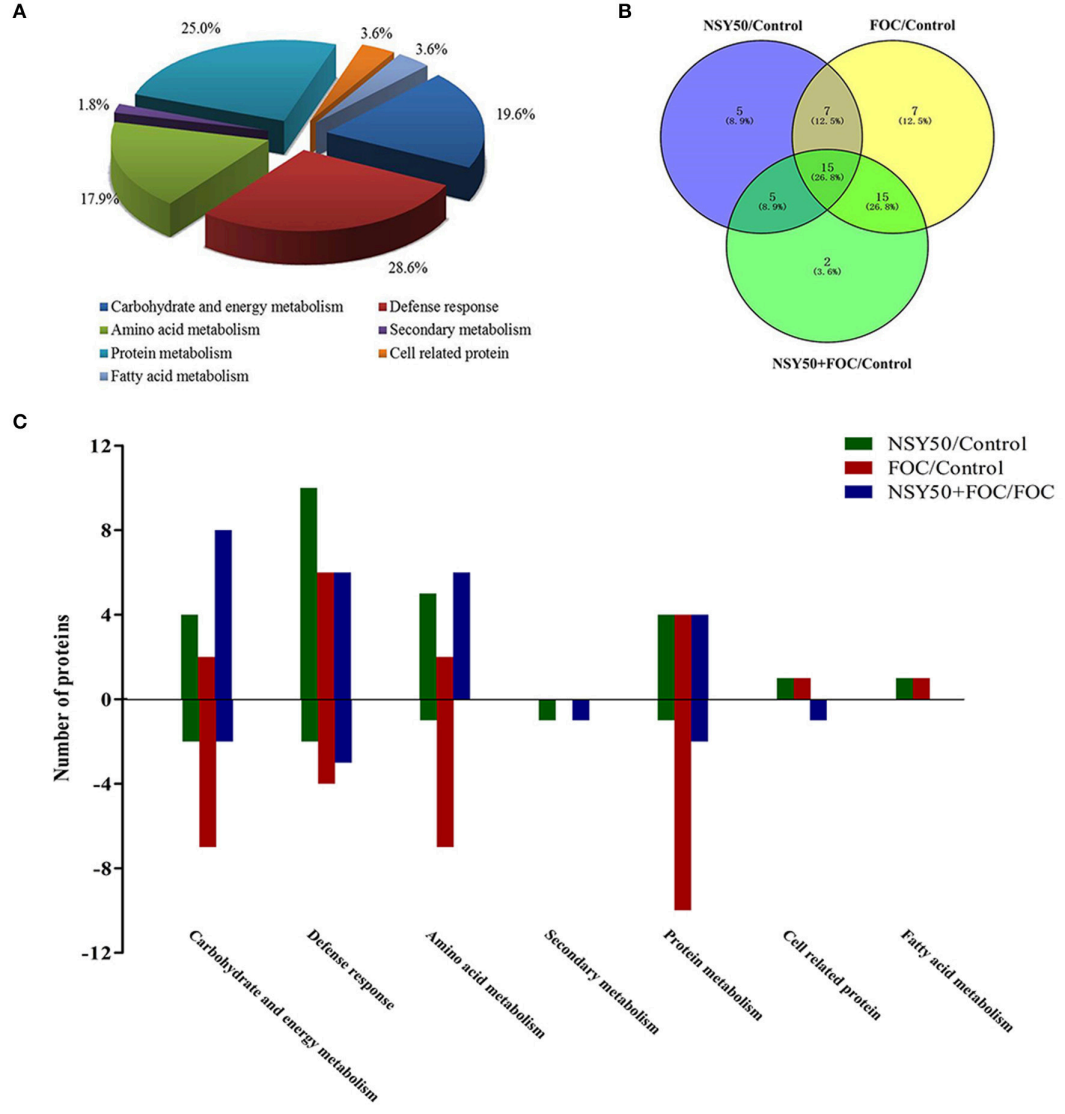
**Distribution of differentially expressed proteins by NSY50 and/or FOC in cucumber roots. (A)** Functional classification and distribution of all 56 differentially expressed proteins. **(B)** Venn diagram showing the number of overlapping proteins that were differentially regulated by NSY50, FOC, and NSY50+FOC compared to the control. **(C)** Functional protein distribution in the compared groups (changes ≥1.5-fold or ≤0.67-fold).

The identified proteins were grouped into seven different categories on the basis of biological processes to which they were related according to the Gene Ontology and Uniprot Protein Knowledgebase (Figure 3A). The categories with a high level of expression variation included the defense response (28.6%), protein metabolism (25.0%), carbohydrate and energy metabolism (19.6%) and amino acid metabolism (17.9%). Among the 56 differentially expressed spots, 44 protein spots were significantly regulated by the pathogen FOC compared to the control (Figure 3B), and there were 16 up-regulated spots and 28 down-regulated spots (Figure 3C). The defense-response-related proteins had the highest up-regulation rate, and the decreased proteins were involved in protein metabolism (10 spots), carbohydrate, and energy metabolism (7 spots), and amino acid metabolism (7 spots). Additionally, 32 protein spots were significantly induced by NSY50 inoculation compared to the control (Figure 3B). Of those spots, 25 were up-regulated, and seven were down-regulated. The defense-response-related proteins (10 spots) and amino acid metabolism (5 spots) were the most highly enriched categories (Figure 3C). However, compared to FOC, 33 proteins had changes, including 24 proteins that increased and 9 proteins that decreased after pre-treatment with NSY50 (NSY50+FOC). The category of carbohydrate and energy metabolism had the highest up-regulation rate, whereas the decreased proteins were distributed among the defense response, protein metabolism, secondary metabolism and cell-related protein categories (Figure 3C).

### Clustering analysis of differentially expressed proteins

**Figure 4:**
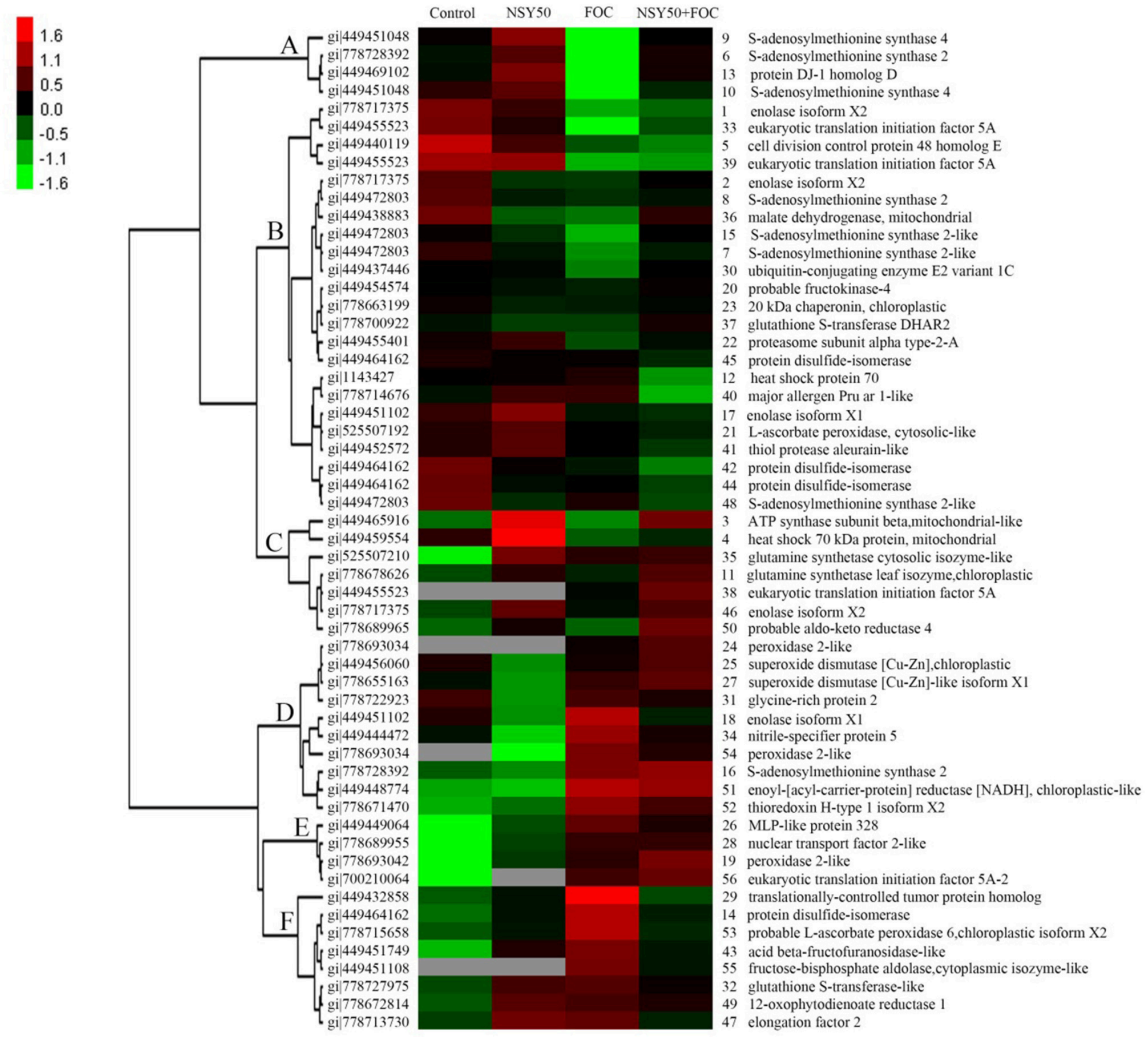
**Hierarchical clustering analysis of the differentially expressed proteins responding to NSY50 and/or FOC**. The fold changes of protein abundance among the four treatments were log2 transformed and delivered to the Cluster and Treeview software. Each row represents individual protein spots and spot numbers, and the protein names are labeled to the right of the corresponding heat maps. Red and green show the higher and lower expression levels, respectively.

To acquire a comprehensive overview of the differentially expressed proteins that were affected by NSY50 under control conditions and FOC stress, hierarchical clustering was performed, and proteins with similar expression patterns were grouped together (Figure 4). Cluster A consisted of 4 proteins (spots 9, 6, 13, and 10) that were up-regulated by NSY50, down-regulated after inoculation with FOC compared to the control, but recovered by the application of NSY50 (NSY50+FOC). Cluster B was composed of 23 proteins that decreased considerably under FOC stress with/without NSY50 inoculation. Most of the identified proteins in this cluster were involved in protein metabolism, carbohydrate and energy metabolism. Cluster C involved 7 proteins that were up-regulated by NSY50 under both control conditions and FOC stress conditions, and these proteins corresponded to defense responses, carbohydrate and energy metabolism, amino acid metabolism, and protein metabolism. Cluster D included 10 proteins that were down-regulated by NSY50 but up-regulated by the inoculation of FOC. Most of these proteins were involved in defense responses. Cluster E contained 4 proteins that were down-regulated by NSY50 but up-regulated by the inoculation of FOC, regardless of whether they were pre-inoculated with NSY50. Cluster F included 8 proteins that increased in abundance when inoculated with NSY50 or FOC alone but were down-regulated in combined inoculation treatment (NSY50+FOC).

### Validation of differentially expressed proteins

**Figure 5:**
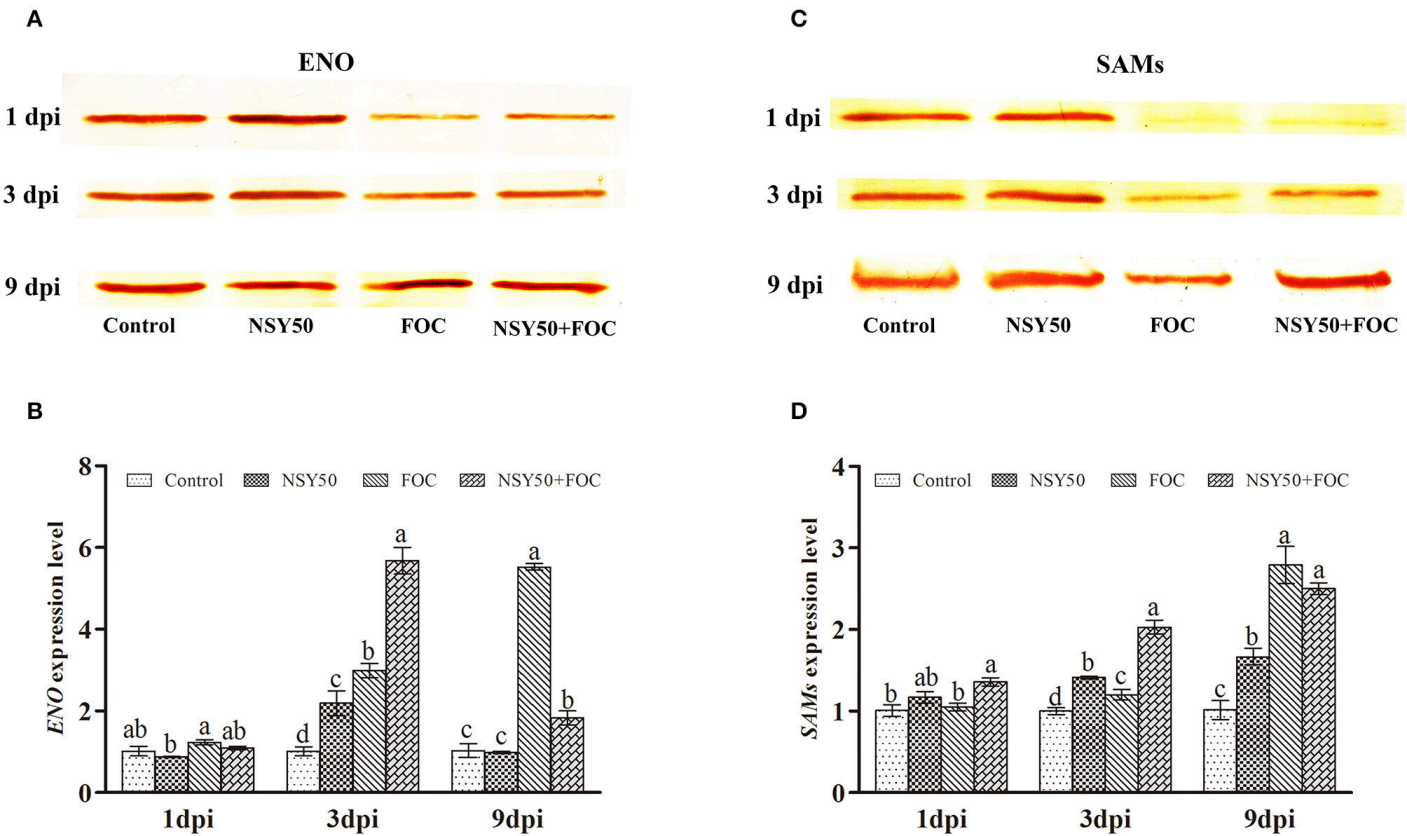
**Western blot and qRT-PCR analysis of the proteins and genes of ENO and SAMs expression levels for the four treatments in the roots of cucumber seedlings challenged with ***F. oxysporum*** at 1, 3, and 9 days post-inoculation (dpi)**. **(A)** Western blot analysis of the ENO expression level for the four treatments over 3 days; **(B)** qRT-PCR analysis the *ENO* gene expression level for the four treatments over 3 days; **(C)** Western blot analysis of the SAMs expression level for the four treatments over 3 days; **(D)** qRT-PCR analysis of the *SAMs* gene expression level for the four treatments over 3 days. Bars marked with dissimilar letters are significantly different according to Duncan's multiple range test (*P* < 0.05). Control, seedlings were cultured in normal nutrient solution; NSY50, control+ *P. polymyxa* NSY50 (2.5 × 10^6^ CFU/mL); FOC, control+ FOC (2.5 × 10^5^ conidia/mL); NSY50+FOC, pre-treated with NSY50 (2.5 × 10^6^ CFU/mL) for 3 days, then challenged with FOC (2.5 × 10^5^ conidia/mL).

As shown in Table 2, five differentially expressed protein spots belonging to ENO and eight SAMs protein spots were successfully identified by MALDI-TOF/TOF MS, and they were all significantly regulated by inoculation with NSY50 and/or FOC. This outcome indicated that ENO and SAMs were considered important plant proteins for coping with pathogen invasion. Therefore, ENO and SAMs were analyzed by western blotting to verify the proteomic data. The result showed a similar tendency, in that the expression of ENO and SAMs were slightly more highly regulated by the inoculation of NSY50 but significantly down-regulated by FOC infection compared to the control. However, the expression of both of these proteins was recovered by pre-treatment with NSY50 (NSY50+FOC) compared to FOC, regardless of whether measurements were taken 1, 3, or 9 days post-inoculation with FOC (Figures 5A,C; Supplementary Figures 2–5). Additionally, the level of SAMs was seriously decreased by FOC inoculation on the first day after inoculation (Figure 5C).

### Expression analysis of several identified protein-related genes

The *ENO* and *SAMs* gene expression levels were then analyzed 1, 3, and 9 days post-inoculation. FOC up-regulated both proteins at all time points compared to the control (Figures 5B,D). The gene expression of *ENO* after NSY50 treatment was significantly lower compared to the FOC treatment. However, *SAMs* showed a contrary tendency in the early stages of inoculation (Figure 5D), and the transcript levels in NSY50+FOC were higher and increased more rapidly compared to those of plants that were inoculated with only FOC (FO C) from 1 to 3 days post-inoculation.

**Figure 6:**
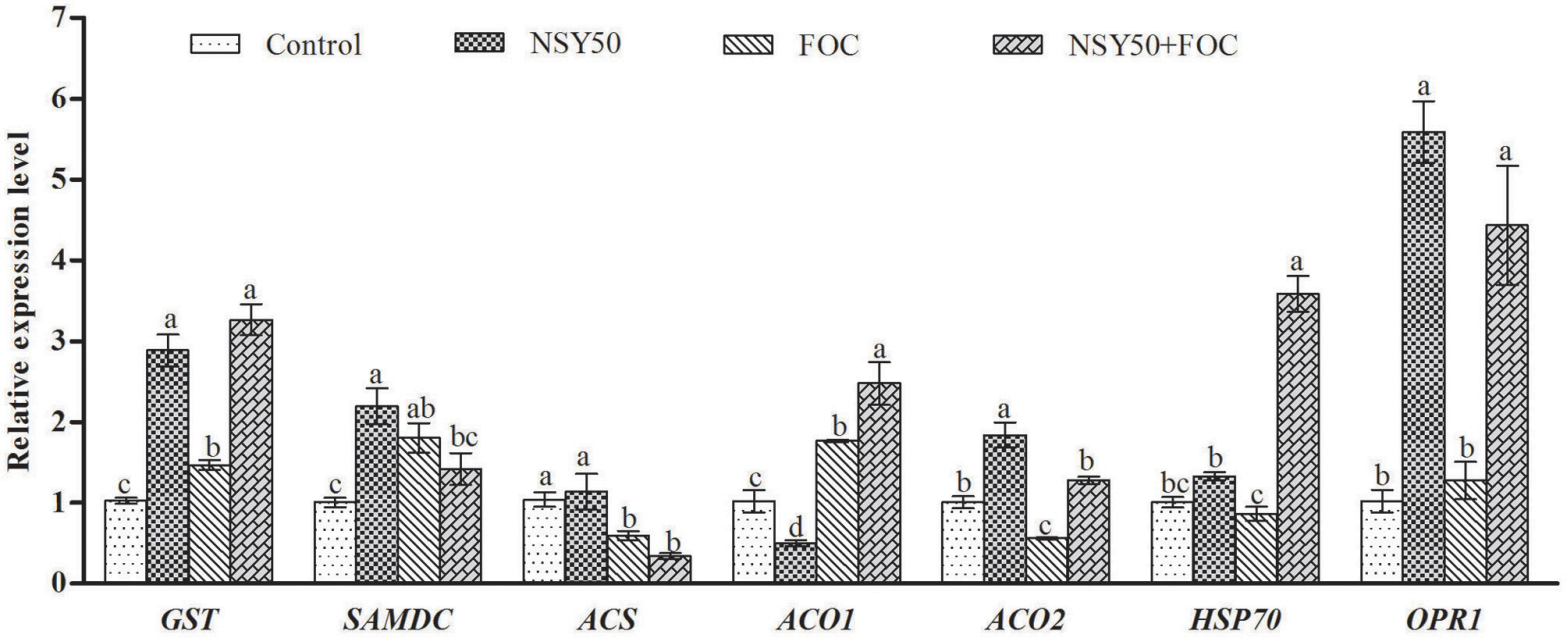
**Effects of NSY50 and/or FOC treatment on transcript analysis of ***ENO, GST, SAMs, SAMDC, ACS, ACO1, ACO2, HSP70***, and ***OPR1*** in the roots of cucumber seedlings**. Each histogram represents a mean ± SE of three independent experiments (*n* = 3). Different letters indicate significant differences between the treatments (*P* < 0.05) according to Duncan's multiple range test. Abbreviations: *ENO*, enolase; *GST*, glutathione S-transferase; *SAMs*, S-adenosylmethionine synthase; *SAMDC*, S-adenosylmethionine decarboxylase; *ACS*, 1-aminocyclopropane-1- carboxylate synthase; *ACO1*, 1-aminocyclopropane-1-carboxylate oxidase 1; *ACO2*, 1-amin ocyclopropane-1-carboxylate oxidase 2; *HSP70*, heat shock 70 kDa protein; *OPR1*, oxophytodienoate reductase 1.

**Figure 7:**
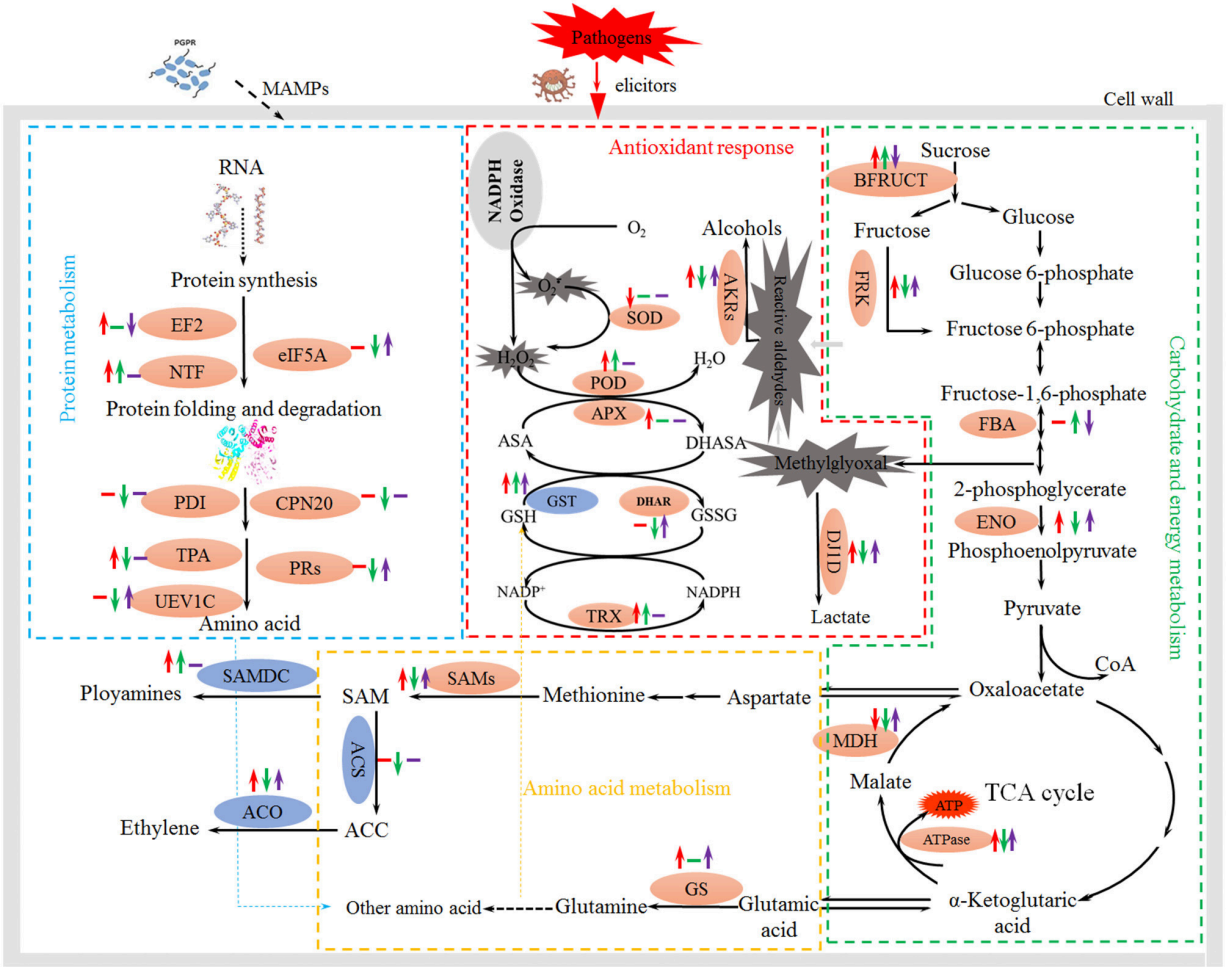
**Schematic presentation of the effects of PGPB and FOC stress on metabolism in cucumber roots**. Changes in protein abundance (marked in red ellipses) and gene expression (blue) were integrated. Red arrows on the life side indicate changes induced by NSY50 compared to the control, green arrows in the middle indicate changes induced by FOC compared to the control, and the purple arrows on the right side indicate changes induced by combined inoculation (NSY50+FOC) conditions. Arrows ↑ or ↓ represent up-regulation or down-regulation, and short lines indicate no change. Abbreviations: BFRUCT, acid beta-fructofuranosidase; FRK, fructokinase; FBA, fructose-bisphosphate aldolase; ENO, enolase; MDH, malate dehydrogenase; ATPase, ATP synthase; SOD, superoxide dismutase; POD, peroxidase; APX, ascorbate peroxidase; GST, glutathione S-transferase; DHAR, glutathione S-transferase DHAR; TRX, thioredoxin; GS, glutamine synthetase; SAMs, S-adenosylmethionine synthase; SAMDC, S-adenosylmethionine decarboxylase; ACS, 1-aminocyclopropane-1-carboxylate synthase; ACO,1-aminocyclopropane-1-carboxylate oxidase; EF2, elongation factor 2; NTF, nuclear transport factor; eIF5A, eukaryotic translation initiation factor 5A; PDI, protein disulfide-isomerase; CPN20, 20 kDa chaperonin; TPA, thiol protease aleurain-like; PRs, proteasome; UEV1C, ubiquitin-conjugating enzyme E2 variant 1C.

Seven other genes related to the identified proteins, including *GST, SAMDC, ACS, ACO1, ACO2, HSP70*, and *OPR1*, were selected and subjected to expression pattern analysis. NSY50 up-regulated almost all of these genes (except *ACS* and *ACO1*) compared to the control (Figure 6). FOC stress up-regulated the expression of *GST, SAMDC* and *ACO1* but decreased the *ACS* and *ACO2* expression. The combined inoculation (NSY50+FOC) treatments up-regulated expression of almost all of these genes (except *SAMDC* and *ACS*) compared with the FOC treatment. We summarize the metabolism pathways that connect the expression of these genes, the proteins related to an antioxidant response, amino acid metabolism, and the EMP-TCA cycle in Figure 7.

## Discussion

Biological control is one of the most beneficial methods because it is both efficient and environmentally friendly for plant protection (Bargabus et al., 2003; Tjamos et al., 2005). Currently, the commercialization of bio-control agents is still limited (Szewczyk et al., 2006) due to complicated mechanisms of bio-control that have yet to be clarified (Niu et al., 2011). *Paenibacillus polymyxa* has been considered a bio-control agent for a wide range of plant pathogens (Dijksterhuis et al., 1999; Ling et al., 2011; Hong et al., 2016). It is an important Bacillus species because of its ability to produce different bioactive compounds with a broad spectrum of activities or induce systemic resistance against a variety of environmental stresses (Kim et al., 2015; Zhou et al., 2016). With the goal of commercializing *P. polymyxa* NSY50 in the future, we analyzed the proteins in cucumber roots that responded to NSY50 under FOC stress conditions to investigate the promotion and protection mechanisms of NSY50. The results of our proteome analysis are discussed below.

### Proteins related to defense

Reactive oxygen species (ROS) have been demonstrated to be involved in symbiotic interactions between plants and microorganisms (Scheler et al., 2013). During pathogen invasion, plants have always suffered from ROS and reactive aldehyde bursts, which often lead to cellular damage (Niu et al., 2011; Gupta et al., 2014; Sengupta et al., 2015; Camejo et al., 2016). Fortunately, plants have developed an antioxidant enzymatic system to scavenge for and collect these toxic compounds. In the present study, a total of 16 spots were identified as defense-related proteins, and most of them were regulated by the inoculation of NSY50 and/or FOC (Table 2, Figure 3C).

Two superoxide dismutases (SOD, spots 25, and 27) were identified and were significantly up-regulated by pre-treatment with NSY50 (NSY50+FOC) compared to FOC treatment. Three peroxidases (POD, spots 19, 24, and 54) with different subunits were identified and showed different accumulation patterns. Regardless of whether they were inoculated with NSY50 or FOC, the expression levels were both up-regulated compared to the control. Additionally, spots 19 and 24 showed expression levels in NSY50+FOC that were higher than the proteins inoculated with FOC alone. Ascorbate peroxidase (APX, spot 21) was also up-regulated by NSY50 (NSY50) compared to the control. It is known that the activation of antioxidant enzymes is a major plant cell method for protection (Youssef et al., 2016). Several studies confirmed the ability of bio-control agents to mitigate the effects of oxidative bursts by increasing the activity of antioxidant enzymes (Israr et al., 2016; Youssef et al., 2016). Thus, the results of the present study indicated that application of NSY50 played an active role in determining the abundance of antioxidant enzymes.

GSTs belong to a family of multifunctional enzymes that play critical roles in protecting tissues from oxidative damage by quenching reactive molecules (An et al., 2016). Previous studies have reported that GSTs can enhance resistance to the virulent bacterial pathogen *Pseudomonas syringae* pv. tomato DC3000 in *Arabidopsis* (Jones et al., 2006) and were increased in response to various hormones, such as salicylic acid, ethylene, methyl jasmonate, and auxin, and to biotic and abiotic stress (Lieberherr et al., 2003; Faltin et al., 2010). Additionally, Kwon et al. (2016) found that four glutathione S-transferases were significantly up-regulated by plant-growth-promoting rhizobacterium *P. polymyxa* E681 in *Arabidopsis* and suggested that the activation of defense-related proteins may the reason for resistance to the fungal pathogen. It was reported that the overexpression of GSTs induced by another PGPR, *P. fluorescens*, had an essential role in the ISR by priming rice plants and protecting cells from oxidative damage (Kandasamy et al., 2009). In the present study, two glutathione S-transferases (GST, spots 32, and 37) were identified. One of them (spot 32) was significantly up-regulated in NSY50, and the other (spot 37) showed expression levels in NSY50+FOC that were much higher than those in FOC. The qRT-PCR analysis also showed that the application of NSY50 significantly up-regulated this gene expression, regardless of whether the plants were inoculated with FOC (Figure 6). This result indicated that NSY50 could increase GST content to remove toxins in cucumber seedlings.

Plant thioredoxins appear to play a fundamental role in plant tolerance of oxidative stress by supplying power to reductases detoxifying lipid hydroperoxides or repairing oxidized proteins (Vieira Dos Santos and Rey, 2006). Thioredoxin H-type 1 isoform X2 (TRX, spot 52) belongs to the thioredoxins family and was up-regulated by the inoculation of NSY50 and/or FOC. This result was consistent with the results of Laloi et al. (2004), who reported that the *Arabidopsis* cytosolic thioredoxin h5 gene was induced by oxidative stress and its response to a pathogen elicitor. In addition, Sun et al. (2010) found that the overexpression of NtTRXh3 (An h-type thioredoxin in tobacco) conferred resistance to Tobacco mosaic virus and Cucumber mosaic virus, both of which had reduced multiplication and pathogenicity in NtTRXh3- overexpressing plants compared to controls. These reductions indicated that TRX participated in defense mechanisms linked to the oxidative bursts resulting from pathogen attack.

In this study, a probable aldo-keto reductase 4 (AKRs, spots 50) was successfully identified, which showed that inoculation with NSY50 significantly up-regulated the expression of aldo-keto reductase (AKRs), with or without FOC. The AKRs superfamily comprises a large number of primarily monomeric protein members that reduce a broad spectrum of substrates, which range from simple sugars to potentially toxic aldehydes (Sengupta et al., 2015). There are a number of interesting reports on the active involvement of plant AKRs in detoxification of stress-induced reactive carbonyls as plant defense systems against biotic stress factors, such as pathogenic attack (Tremblay et al., 2010; Santos et al., 2012; Xu et al., 2013). This may be the first report on a type of protein induced by the PGPR that actively contributes to defense against biotic stressors.

Other defense response proteins, such as heat shock protein 70 (HSP70, spots 4 and 12) and MLP-like protein 328 (MLP328, spot 26), were also identified in this study. HSP70 can be induced by various environmental stresses, such as cold, salt, high temperature, and hypoxic stress, and this protein often acts as a molecular chaperone (Xu et al., 2011; Li et al., 2013b; Yuan et al., 2016) to prevent the irreversible aggregation of other proteins and regulate the folding and accumulation of proteins (Feder and Hofmann, 1999). In this study, it was observed that NSY50 enhances the expression of HSP70 (spot 4) by 3.31-fold compared to the control. Whereas, FOC significantly decreased HSP70 expression both under control conditions and pre-treatment with NSY50 (Table 2), the combined inoculation (NSY50+FOC) showed much higher up-regulation than FOC, although there was no significant difference. The transcription of *HSP70* showed a significant increase in NSY50+FOC compared to inoculation with FOC alone (Figure 6). This result indicates that the PGPG are capable of enhancing the expression of this important protein. This result was consistent with the research of Li et al. (2013b), who reported that PGPR *P. putida* UW4 significantly enhanced the level of plant heat shock proteins under hypoxic stress to improve stress resistance. The role of the MLP-like protein was unclear, and it was hypothesized to be a physiological defense protein that responded to stress (Fukao et al., 2009; An et al., 2016). However, in this study, MLP328 was significantly up-regulated, with a 3.13–3.68-fold increase following the inoculation of NSY50 and/or FOC compared to the control. The mechanism of this type of physiological defense protein needs further study.

### Protein metabolism

Of the differentially accumulated proteins, 14 were identified as responding to FOC and/or NSY50 treatments; these responses were attributed to protein metabolism, and the proteins could be divided into five functional groups (Table 2). The first group includes those involved in protein folding and assembly, including four protein disulfide-isomerase (PDI, spots 14, 42, 44, and 45) and a 20 kDa chaperonin (CPN20, spot 23). Both the NSY50 and FOC treatments enhanced the expression of PDI (spot 14) by 1.90–2.42-fold compared to the control. Several studies have shown that the overexpression of PDI in plants that are challenged with pathogens has been thought to be related to the correct folding of defense proteins (Gruber et al., 2007; Palomares-Rius et al., 2011; Cipriano et al., 2015). However, the remaining three PDI proteins (spots 42, 44, and 45) were significantly decreased by pathogen attack (inoculated with FOC). This effect may be due to the stresses that were inhibited expression of this type of protein, which was consistent with previous reports (Yuan et al., 2016). However, another report showed that the application of PGPR recovered the expression level of this protein under stress conditions (Li et al., 2013b). The CPN20 (spot 23), which was among the most-represented proteins that emphasize the impact of stress on post-translation modification/ modification machinery (Zhang et al., 2015) showed similar results when inoculated with FOC.

The second group included three spots of eukaryotic translation initiation factor 5A (eIF-5A, spots 33, 38, and 39), one eukaryotic translation initiation factor 5A-2 (eIF-5A2, spot 56) and one elongation factor 2 (EF2, spot 47), which are involved in the initiation and elongation stage of mRNA translation and protein synthesis (Thompson et al., 2004; Yuan et al., 2016). Overall, the FOC inoculation significantly decreased the eIF-5A expression levels in cucumber seedling roots, whereas pre-treatment with NSY50 (NSY50+FOC) enhanced the expression of eIF-5A. However, the eIF-5A2 protein had a higher accumulation after FOC inoculation conditions both with and without NSY50 compared to non-stress conditions. The protein eIF-5A2 was reported to likely be involved in the regulation of apoptotic cell death (Feng et al., 2007). EF2 was significantly up-regulated, with a 2.70-fold increase after inoculation with NSY50 compared to the control, whereas inoculation with FOC showed only a slight increase. These results indicated that the PGPR could balance ribosomal particles and protein synthesis, which was consistent with previous reports (Li et al., 2013b).

The third group included proteasome subunit alpha type-2-A (PRs, spot 22) and thiol protease aleurain-like (TPA, spot 41), which are involved in protein degradation. The proteasome was assigned a precise role in the degradation of the oxidized proteins generated by many stresses, which avoided the inhibition of the plant's metabolic pathway (Polge et al., 2009). In this study, infection with FOC down-regulated the expression level of TPA, and the plants metabolic pathway may have been damaged by this destructive fungal pathogen, which was consistent with the TEM result. The PRs significantly decreased under FOC stress but were remarkably up-regulated by inoculation with NSY50, which indicated that the degradation of proteins with oxidative damage was enhanced (An et al., 2016). The fourth group was protein transport and included one protein called nuclear transport factor 2-like (NTF2, spot 28). The NTF2 facilitated protein transport into the nucleus (Zhao et al., 2006). Both inoculation with NSY50 and FOC were significantly up-regulated the expression of this protein. The ubiquitin-conjugating enzyme E2 variant 1C (UEV1C, spot 30) belonged to the fifth group, protein modification, and it participates in protein modification by catalyzing the covalent attachment of ubiquitin to proteins (Yuan et al., 2016). In this study, FOC significantly decreased the expression of UEV1C, whereas pre-treatment with NSY50 (NSY50+FOC) enhanced its expression. These results suggest that the application of NSY50 alleviated the damage to protein metabolism that was induced by *F. oxysporum*.

### Carbohydrate and energy metabolism

The proteins related to carbohydrate and energy metabolism, particularly the proteins involved in the glycolysis (EMP) and tricarboxylic acid (TCA) cycle were significantly changed and are shown in Table 2. A total of 11 identified proteins in response to two strains were found and were divided into four functional groups. The first group focused on glycolysis and included five spots with enolase isoform (ENO, spots 1, 2, 17, 18, and 46), protein DJ-1 homolog D (DJ1D, spot 13), probable fructokinase-4 (FRK, spot 20) and fructose-bisphosphate aldolase (FBA, spot 55). The others were involved with the tricarboxylic acid cycle, including malate dehydrogenase (MDH, spot 36), sucrose metabolism protein acid beta-fructofuranosidase-like (BFRUCT, spot 43), and energy metabolism protein ATP synthase (ATPase, spot 3). Almost all of them were down-regulated by FOC and recovered by treatment with NSY50 (NSY50+FOC), except FBA and BFRUCT.

Enolases, known as 2-phospho-D-glycerate hydrolases, are one of the most important enzymes in glycolysis. They catalyze the dehydration of 2-phosphoglycerate into phosphoenolpyruvate. Five spots were identified as enolases in this study, and western blotting was used to verify proteomic data (Table 2, Figure 5A; Supplementary Figures 2, 3) and showed a similar tendency, regardless of whether measurements were taken 1, 3, or 9 days post-inoculation with the pathogen FOC. The gene expression showed a reverse trend, and inoculation with FOC significantly up-regulated its gene expression (Figure 5B). Interestingly, protein abundance significantly recovered along with inoculation time (Figure 5A). Several studies reported that both the ENO protein and the gene expression increased under salt stress and attempted to generate more energy to cope with the stress (Ndimba et al., 2005; Zhong et al., 2015; An et al., 2016; Yuan et al., 2016).

The DJ-1D protein (DJ1D, spot 13), which was reported to have several functions, including functioning as an antioxidant (Taira et al., 2004), is a redox-dependent chaperone (Krebiehl et al., 2010) and can convert glyoxals to carboxylic acids (Kwon et al., 2013). Glyoxals are reactive 2-oxoaldehydes that are found in cells under various stress conditions (Thornalley, 1996). In this study, DJ1D was significantly up-regulated, with 4.25- and 2.28-fold increases after inoculation with NSY50 and/or FOC, respectively.

Li et al. (2013b) reported that the application of PGPR *P. putida* UW4 adjusted the EMP-TCA metabolism under hypoxic stress. In contrast, Kwon et al. (2016) found that some of the proteins related to the glycolysis pathway, such as FBA and pyruvate dehydrogenase complex component E2, were significantly up-regulated by the PGPR strain *P. polymyxa* E681 in *Arabidopsis* roots. Carbohydrate metabolism provides the plant with required carbon, which is critical for the production of new tissues, and also can have profound effects on plant growth through modulation of cell division and expansion (Eveland and Jackson, 2012; Kwon et al., 2016). In this study, we found that the application of *P. polymyxa* NSY50 increased the abundance of FRK, ENO, BFRUCT, and ATPase compared to control conditions. Additionally, NSY50 partially recovered the ENO, FRK, DJ1D, MDH, and ATPase expression, which had been decreased by FOC. These results suggested that the application of NSY50 could promote EMP-TCA pathway activity. Presumably, these results imply there was an enhancement in energy generation and carbohydrate production to promote plant growth and resist stress damage.

### Amino acid metabolism

Ten spots were identified as proteins involved in amino acid metabolism. Eight S-adenosylmethionine synthases (SAMs, spots 6, 7, 8, 9, 10, 15, 16, and 48) comprising different subunits were identified and markedly decreased (except spot 16) in response to FOC infection. SAMs catalyze the biosynthesis of S-adenosylmethionine (SAM) from methionine and ATP, and they are active in the biosynthesis of lignin, glycine betaine and polyamines (Fontecave et al., 2004). These proteins can be induced by various environmental stresses, such as cold, salt, drought stress and even pathogen infection (Li et al., 2013a; Huang et al., 2016), and most of them were down-regulated by stress (Wang et al., 2016; Yuan et al., 2016). This outcome was consistent with the results showing that the inoculation of FOC significantly decreased SAMs expression (Table 2, Figure 5C; Supplementary Figures 4, 5). However, pre-treatment with NSY50 up-regulated SAMs abundance both under control and pathogen infection conditions. The accumulation of SAMs in plants has been shown to be related to the enhance tolerance to various kinds of environmental stresses. Overexpression of *suadea salsa* SAMs gene promoted salt tolerance in transgenic tobacco (Qi et al., 2010). An increase in production of SAMs was observed in cold stressed rice (Cui et al., 2005), mechanically wounded *Phaseolus lunatus* (Arimura et al., 2002), salt-stressed barley (Witzel et al., 2009), and in response to cotton worm feeding in soybean (Fan et al., 2012). Hence, the remarkable expression of SAMs by NSY50 suggested that plant might promote SAM-dependant metabolism through synthesize more SAM to further enhance the synthesis of new proteins and stress tolerance.

Glutamine synthetase (GS) is a key enzyme in plant N assimilation that catalyzes the combination of ammonia and glutamate into glutamine (Nam et al., 2011; Rogić et al., 2015). Two types of GS (spots 11 and 35) were identified and showed different accumulation patterns. However, they were both up-regulated by NSY50, regardless of whether the plants were inoculated with FOC. This result suggested that the application of NSY50 could lead to a high accumulation of amino acid metabolism, and this was probably one way in which NSY50 promoted plant growth and intensified resistance to pathogen infection for cucumber seedlings.

### Fatty acid metabolism proteins

Jasmonic acid is a signaling compound that influences multiple cellular functions and plays crucial roles in the signaling network regulating the development of induced resistance, including systemic acquired resistance (SAR) and induced systemic resistance (Glazebrook, 2001). Niu et al. (2011) found that PGPR *Bacillus cereus* AR156 induced ISR to *Pseudomonas syringae* pv. *tomato* DC3000 in *Arabidopsis* via the simultaneous activation of salicylic acid-, jasmonic acid-, and ethylene-dependent signaling pathways. In the present study, 12-oxophytodienoate reductase 1 (spot 49), which is involved in jasmonate biosynthesis, was identified to be induced by both NSY50 and FOC inoculation. Additionally, NSY50 treatment showed a 2.02-fold increase in abundance compared to FOC. A good correlation was observed between this protein and transcript abundance, as etermined by qPCR analysis (Figure 6). This outcome indicated that *P. polymyxa* NSY50 may stimulate the jasmonate signal transduction network.

Enoyl-[acyl-carrier-protein] reductase [NADH] (ACP, spot 51), which is a subunit of the fatty acid synthase complex that catalyzes the *de novo* synthesis of fatty acids (Mou et al., 2000), was significantly up-regulated by inoculation with FOC. It was recently reported that ACP was sensitive to the diphenyl ether cyperin produced by certain pathogenic fungi in the tissues of infected plants, which likely contributed to the virulence of these disease agents (Dayan et al., 2008). The role of the ACP during pathogen infection remains unclear, and further study is required.

### Secondary metabolism and cell-related proteins

The NSY50 was also found to regulate proteins involved in other metabolic pathways, such as secondary metabolism (spot 34) and cell-related protein (spots 5 and 29), which indicated that NSY50 regulated various metabolic pathways to counteract FOC infection. The mechanism of action will need to be studied in future investigations.

## Conclusion

To the best of our knowledge, this is the first proteomic-based research to focus on the interactions between cucumber plants, the pathogen *F. oxysporum*, and PGPR strain *P. polymyxa* NSY50. A total of 56 protein spots were identified with a change more than 1.5-fold or less than the 0.67-fold in the protein abundance ratio in cucumber seedling roots after inoculation with *P. polymyxa* NSY50 and/or FOC. The majority of these proteins were related to defense responses, protein metabolism, carbohydrate and energy metabolism and amino acid metabolism. These proteins might work cooperatively to enhance resistance to FOC attack and keep plant growth on a mostly even keel. Our results showed that the improved plant growth and defenses by *P. polymyxa* NSY50 might be associated with the following processes: (i) activation of stress defense-related proteins by stress injuries that are alleviated by NSY50; (ii) NSY50 stimulating protein synthesis and degrading damaged proteins induced by FOC; (iii) up-regulation of proteins in the EMP-TCA route to provide more carbohydrates and energy to promote plant growth and resist debilitation; or (iv) adjusting the plant amino acid metabolism to increase biomass of the plant and regulate other metabolic pathways, such as jasmonic acid, to enhance resistance to FOC. This research enriched our understanding of the mechanisms of how PGPR mediates the stress response in plants and promotes protection from pathogen infection.

## Author contributions

We thank the numerous individuals who participated in this research. SG designed the study and guided the research. ND and LS wrote the main manuscript text and performed the experiments. YY prepared all the figures and performed some of the experiments. BL contributed new reagents and analytical tools. SS and JS edited this manuscript. All authors reviewed and approved the manuscript.

## Funding

This work was financially supported by the National Natural Science Foundation of China (No. 31672199, No. 31471869, and No. 31401919), the China Agriculture Research System (CARS-25-C-03), and the Priority Academic Program Development of Jiangsu Higher Education Institutions (PAPD).

### Conflict of interest statement

The authors declare that the research was conducted in the absence of any commercial or financial relationships that could be construed as a potential conflict of interest.
