# Supplementary figures and images for: Proteomic Analysis Reveals the Positive Roles of the Plant-Growth-Promoting Rhizobacterium NSY50 in the Response of Cucumber Roots to Fusarium oxysporum f. sp. cucumerinum Inoculation
Source: Front Plant Sci. 2016 Dec 14;7:1859. doi: 10.3389/fpls.2016.01859 (PMC5155491; doi:10.3389/fpls.2016.01859)

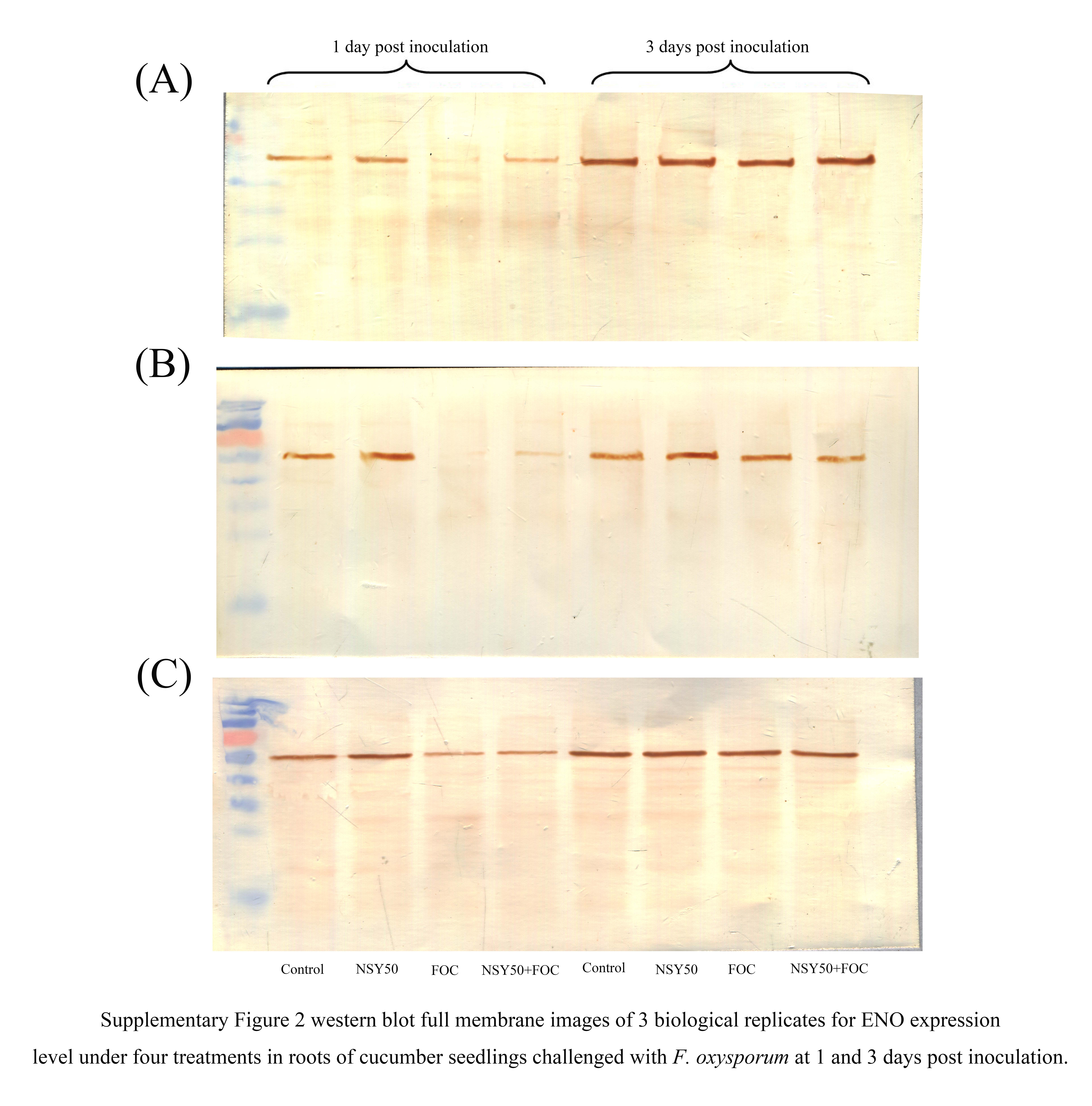

Supplement: Supplementary file 2 [file Image2.JPEG]

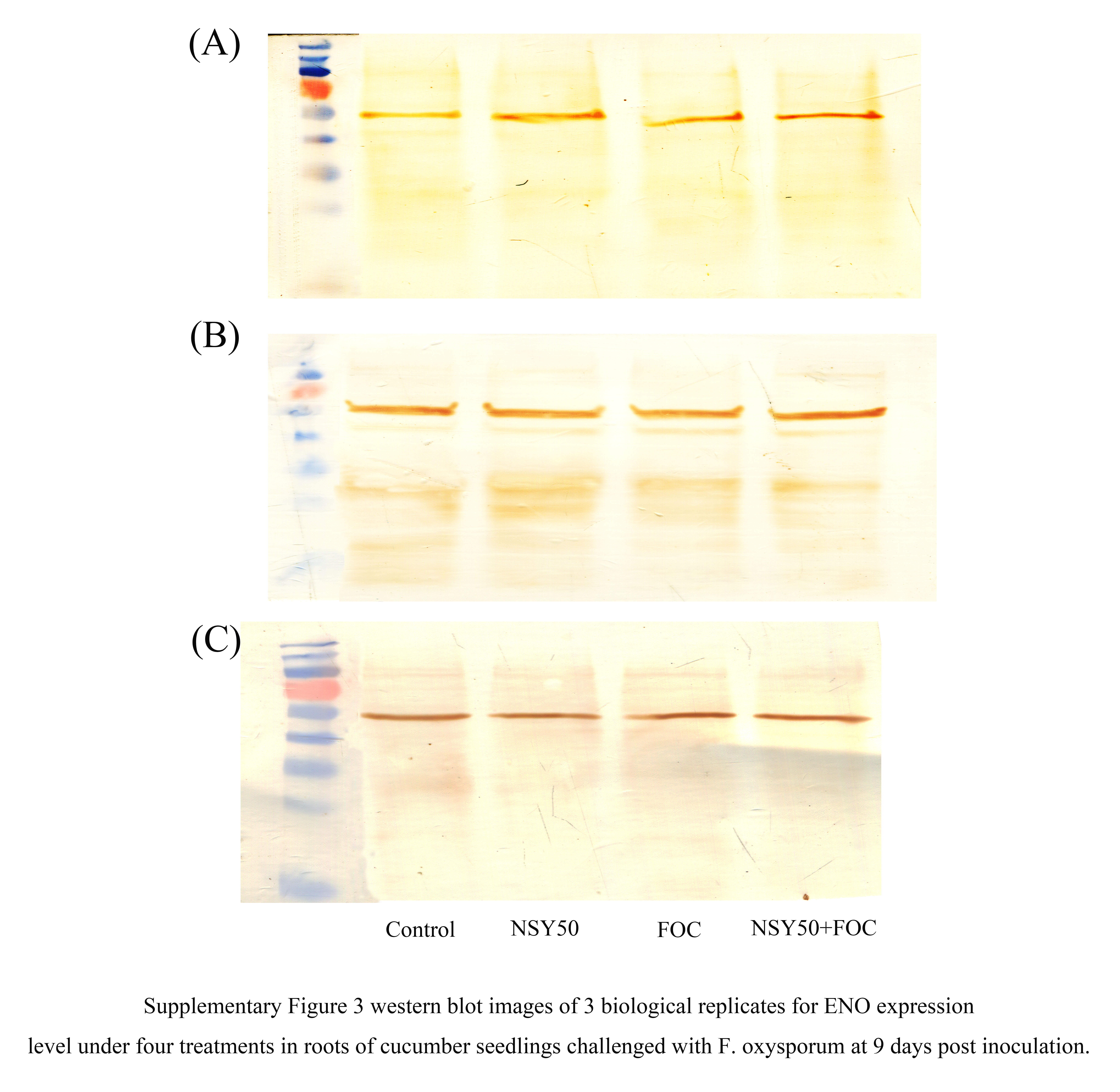

Supplement: Supplementary file 3 [file Image3.JPEG]

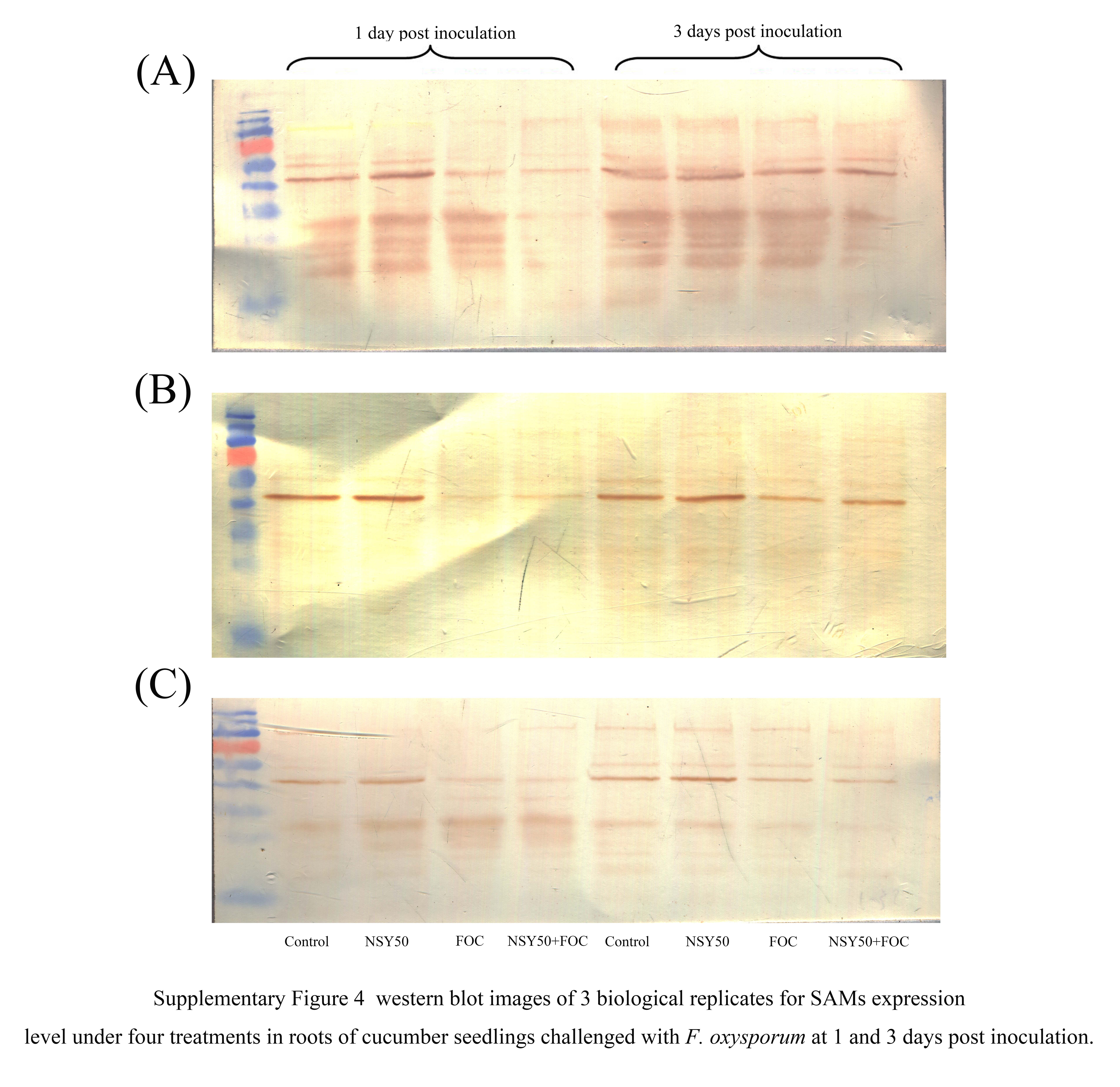

Supplement: Supplementary file 4 [file Image4.JPEG]

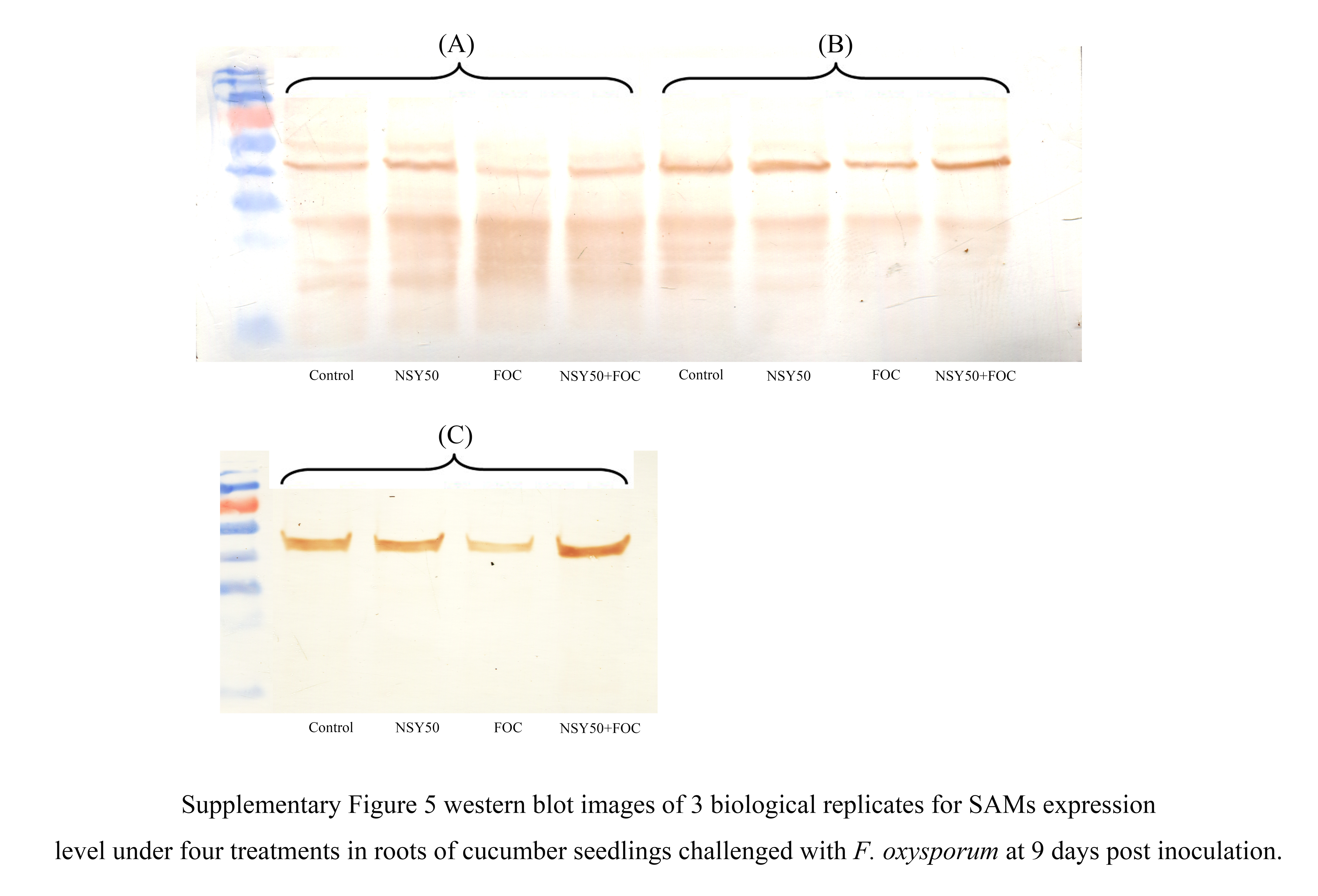

Supplement: Supplementary file 5 [file Image5.JPEG]
