# Supplementary material for: Proteomic Analysis Reveals the Positive Roles of the Plant-Growth-Promoting Rhizobacterium NSY50 in the Response of Cucumber Roots to Fusarium oxysporum f. sp. cucumerinum Inoculation
Source: Front Plant Sci. 2016 Dec 14;7:1859. doi: 10.3389/fpls.2016.01859 (PMC5155491; doi:10.3389/fpls.2016.01859)
Supplement: Supplementary file 6 [file Table1.DOC]

*Supplementary Material*

**Proteomic analysis reveals the positive roles of plant-growth promoting rhizobacterium NSY50 in cucumber roots response to *Fusarium oxysporum* f. sp. *cucumerium* inoculation**

Nanshan Du^1,a^, Lu Shi^1,a^, Yinghui Yuan^1^, Bin Li^3^, Sheng Shu^1,2^, Jin Sun^1,2^, Shirong Guo^1,2^*

**^*^ Correspondence:**

Shirong Guo

srguo@njau.edu.cn

**Supplementary Table 1**

Sequences of gene-specific primers in quantitative real-time polymerase chain reaction (qRT-PCR) analysis

| Gene family | Gene ID | Forward primer (5`- 3`) | Reverse primer (5`-3`) |
| --- | --- | --- | --- |
| ENO | Csa011975 | ATTCGCTCCTAACATTCA | ACATCCATTCCCAAC |
| GST | Csa017734 | TCTCTTGCCTTCTCATCCTT | TGCTCTTCTCCCTTGGTAG |
| SAMs | Csa001882 | AGAGCCACTATCCGTTTTCG | AATCTGGGTCGTCCCTTCCG |
| SAMDC | Csa015017 | GATTGTGAGGGTGCTG | CGACCTTGGAGATGAG |
| ACS | Csa016131 | TTCATTCCATTGCTCAGA | TTAGTTGTTACCTCCTTGTT |
| ACO1 | Csa007981 | GGTTGAGAAGATGACAAGA | AGATGGCGTAAGAAGAAG |
| ACO2 | Csa023227 | CGTCGTAACAGAAGTCAA | TGTAATCGTCGGATAGTTC |
| HSP70 | Csa010385 | ATTGTGCTATTGGATGTG | TTGAGGTAGGAAGTGTAG |
| OPR1 | Csa006042 | GCCATCTTGTATTACTCTC | CATATCCCAGGTGTATCA |

*Note: ENO*, enolase; *GST*, glutathione S-transferase; *SAMs*, S-adenosylmethionine synthase; *SAMDC*, S-adenosylmethionine decarboxylase; *ACS*, 1-aminocyclopropane -1-carboxylate synthase; *ACO1*, 1-aminocyclopropane-1-carboxylate oxidase 1; *ACO2*, 1-amin ocyclopropane-1-carboxylate oxidase 2; *HSP70*, heat shock 70 kDa protein; *OPR1*, oxophytodienoate reductase 1.
